# Evaluation of a Novel Approach for Reducing Emissions of Pharmaceuticals to the Environment

**DOI:** 10.1007/s00267-016-0728-9

**Published:** 2016-06-24

**Authors:** Thomas G. Bean, Ed Bergstrom, Jane Thomas-Oates, Amy Wolff, Peter Bartl, Bob Eaton, Alistair B. A. Boxall

**Affiliations:** 1 Environment Department, University of York, York, YO10 5DD UK; 2 Centre of Excellence in Mass Spectrometry and Department of Chemistry, University of York, York, YO10 5DD UK; 3 PyroPure Ltd, Unit 58 Woolmer Trading Estate, Bordon, Hampshire, GU35 9QF UK; 4 Department of Environmental Science and Technology, University of Maryland, Maryland, MD 20742 USA

**Keywords:** Pharmaceutical waste, Thermal decomposition, Pyrolysis–gasification, Stewardship, Take-back strategy, Antimicrobial resistance

## Abstract

Increased interest over the levels of pharmaceuticals detected in the environment has led to the need for new approaches to manage their emissions. Inappropriate disposal of unused and waste medicines and release from manufacturing plants are believed to be important pathways for pharmaceuticals entering the environment. In situ treatment technologies, which can be used on-site in pharmacies, hospitals, clinics, and at manufacturing plants, might provide a solution. In this study we explored the use of Pyropure, a microscale combined pyrolysis and gasification in situ treatment system for destroying pharmaceutical wastes. This involved selecting 17 pharmaceuticals, including 14 of the most thermally stable compounds currently in use and three of high environmental concern to determine the technology’s success in waste destruction. Treatment simulation studies were done on three different waste types and liquid, solid, and gaseous emissions from the process were analyzed for parent pharmaceutical and known active transformation products. Gaseous emissions were also analyzed for NOx, particulates, dioxins, furans, and metals. Results suggest that Pyropure is an effective treatment process for pharmaceutical wastes: over 99 % of each study pharmaceutical was destroyed by the system without known active transformation products being formed during the treatment process. Emissions of the other gaseous air pollutants were within acceptable levels. Future uptake of the system, or similar in situ treatment approaches, by clinics, pharmacists, and manufacturers could help to reduce the levels of pharmaceuticals in the environment and reduce the economic and environmental costs of current waste management practices.

**Electronic supplementary material:**

The online version of this article (doi:10.1007/s00267-016-0728-9) contains supplementary material, which is available to authorized users.

## Introduction

Active pharmaceutical ingredients (APIs) have been shown to persist in ground, surface, and drinking waters, and have been detected in these water bodies around the world; there is growing concern that these residues can adversely impact the health of ecosystems (Musson and Townsend 2009). It has generally been assumed that excretion by treated individuals is the main source of pharmaceuticals in the environment (Ort et al. 2010). However, there is now increasing recognition that other pathways of exposure such as emissions from manufacturing and the inappropriate disposal of unused medicines could be contributing to the problem (Tong et al. 2011; Daughton and Ruhoy 2009; Daughton 2003; Daughton and Ternes 1999; Larsson 2014; Seehusen and Edwards 2006; Musson et al. 2007).

Medicines can go unused for various reasons including changes in dosage, discontinuation due to side effects, or because the product has reached its expiry date (Boxall et al. 2014). Across several western countries, it has been estimated that anywhere between 3 and 65 % of prescribed pharmaceuticals are not used by the patient (Musson and Townsend 2009; Braund et al. 2009; Seehusen and Edwards 2006). The majority of these unused medicines will be disposed of down the toilet or sink to the sewerage system, or in the household waste to landfill (Vellinga et al. 2014; Glassmeyer et al. 2009). In some regions, these two pathways of disposal are the routes that are recommended to the public (Musson et al. 2007). A percentage of the pharmaceuticals released to the sewerage system can pass through wastewater treatment plants (WWTPs) to receiving surface waters, thus adding to the environmental load. APIs sent to landfills can leach through the site and contaminate groundwater (Musson and Townsend 2009; United States Geological Survey (USGS) 2014; Rodríguez-Navas et al. 2013; Heim et al. 2004; Schwarzbauer et al. 2002; Barnes et al. 2004; Eckel et al. 1993; Holm et al. 1995). As landfill leachate is often transported to WWTPs, APIs sent to landfill can also find their way into rivers and streams (Lubick 2010). Disposal of unused pharmaceuticals or pharmaceutically contaminated waste to landfill could also pose a risk to wildlife which scavenge off these sites, as demonstrated by the poisonings of avian scavengers by sodium pentobarbital contained in euthanized animal carcasses (Langelier 1993; Thomas 1999; Russell and Franson 2014; Boehringer 2004).

From an environmental perspective, the safest way to dispose of unused medicines is to return them to the pharmacy as part of ‘take back’ strategies, where they can be collected and sent to be destroyed in a hazardous-waste high-temperature incinerator (Boehringer 2004; Smith 2002). High-temperature incinerators are also used to treat waste material from pharmaceutical manufacturing plants, e.g., contaminated overalls, batches of APIs that do not meet quality standards, and waste from cleaning machines. Take-back strategies are in operation in a number of regions, although the level of participation in these schemes varies. Data collected in some of the most recent studies into the use of take-back strategies found participation was highest in Sweden with 43 % of people surveyed having returned unused medicines to the pharmacy in the last 12 months (Persson et al. 2009; Tong et al. 2011). Participation was lowest in the USA with only 1.4–5.9 % (Glassmeyer et al. 2009; Kotchen et al. 2009). A recent study in York (UK) found that 17 % of people returned unused medicines to the pharmacy (Williamson and Boxall 2014, unpublished data) which is similar to the 22 % obtained for a UK-wide study a decade previously (Bound and Voulvoulis 2005). These data suggest there is great spatial variability in take-back participation, but in the UK at least, there has not been much change over time.

However, take-back strategies can prove economically costly, as the waste often has to be transported long distances to hazardous-waste incineration facilities. For waste containing controlled substances, the transport has to be secure, adding additional costs. In the UK, for example, there are presently only 22 high-temperature incinerators (DEFRA 2013), meaning that some high value wastes have to be transported hundreds of miles across the country for disposal. Take-back strategies also have an environmental cost e.g., emissions of greenhouse gases during transport to the treatment facility and emissions from the treatment process itself. The availability of in situ treatment systems for waste APIs would reduce disposal costs by eliminating transportation (cost and associated CO_2_ emissions) and associated security costs when wastes contain drugs with street value. Thus in situ waste treatment for pharmaceutical wastes would potentially make take-back strategies economically viable for a greater number of pharmacists and manufacturers. One potential in situ approach is to use combined pyrolysis and gasification treatment technologies. These technologies have the potential to improve environmental compliance by reducing the amount of solid waste produced, effectively destroying the air pollutants generated during the treatment process while simultaneously enabling on-site energy recovery in a way that earlier, separate pyrolysis or gasification units or incinerators cannot (Malkow 2004).

A new microscale pyrolysis–gasification waste treatment technology (hereafter referred to as PGWTS, Pyrolysis–gasification waste treatment system), which could be suited for treatment of chemically contaminated wastes, has recently been developed by Pyropure Ltd (Hampshire, UK). Each unit is about the size of a domestic chest freezer. At the time of this study, up to 8 kg of waste could be treated in each run lasting 3–4 h, but the latest model will treat 16 kg in the same time period. Materials other than glass and metal are reduced to less than 1 % of the initial volume by the end of the run. A waste bag or clinical waste bin can be loaded at one end of the unit into a sealed ‘bin.’ The ‘bin’ part of the PGWTS unit is then heated up by electrical elements (controlled automatically by software) in the absence of air, reaching temperatures in excess of 550 °C (up to 700 °C). Processes of pyrolysis break the waste down to a blackened char. The off-gases are treated by a catalytic converter. On completion of the treatment, the chamber is rinsed by water which then drains to the sewerage system.

In situ PGWTS, like Pyropure, and other new treatment methods, could play an important role in controlling the emissions of APIs to the natural environment (Online Resource 1). However, before these systems are used in the management of pharmaceutically contaminated wastes, it is important that they are shown to be effective at treating APIs. In this paper, we describe the results of the first ever study to evaluate the use of a PGWTS for decomposing APIs in a range of representative waste matrices. As transformation products from treatment processes can pose a risk to the environment (Boxall et al. 2004), we also explored the potential for known active transformation products of the APIs to be formed during the treatment process, using high-resolution mass spectrometry (Fourier transform ion cyclotron resonance mass spectrometry). While the focus of the paper is on assessment of the Pyropure system as an example of a PGWTS, the assessment used could be applied to other new treatment methods for API-containing wastes in the future.

## Materials and Methods

### Selection of Test Pharmaceuticals

**Fig. 1:**
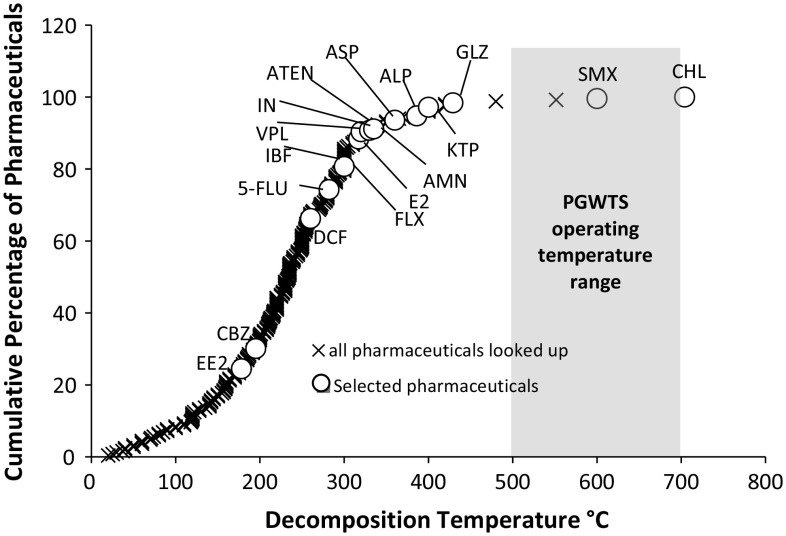
Cumulative percentage of pharmaceuticals looked up in literature review against decomposition temperature (°C). The decomposition temperatures of the 17 pharmaceuticals run through a Pyrolysis–gasification waste treatment system (PGWTS) are marked by white circles and all pharmaceuticals (out of the 600 we looked up) for which we found a decomposition temperature are marked with black crosses. The *white circles* represent the maximum decomposition temperature quoted in the literature for chloramphenicol (CHL), sulfamethoxazole (SMX), gliclazide (GLZ), ketoprofen (KTPF), allopurinol (ALPL), amantadine (AMN), atenolol (ATEN), estradiol (E2), indomethacin (IND), verapamil (VPL), fluoxetine (FLX), ibuprofen (IBF), 5-fluorouracil (5-FLU), diclofenac (DCF), carbamazepine (CBZ), and ethinylestradiol (EE2). The *gray box* represents the typical temperature range in which the PGWTS developed by Pyropure operate. Patient usage (based on NHS prescription cost analysis and over the counter availability (National Health Service (NHS) 2013) and toxicity were also considered when selecting pharmaceuticals, meaning a pharmaceutical with higher toxicity and or usage (E2 and CBZ) was selected over pharmaceuticals with low usage or toxicity e.g., pioglitazone hydrochloride and glimepiride (*two black crosses* between GLZ and SMX)

There are over 4000 APIs in use in Europe and it would be a mammoth task to explore the treatability of all of these molecules (Monteiro and Boxall 2010). As pyrolysis is a thermal-based process, we adopted an approach where the most thermally resistant pharmaceuticals were identified and tested. The assumption being that, if the treatment approach could be shown to work for these, then it should be able to treat all organic APIs. We attempted to obtain thermal decomposition data for the top 300 most highly used APIs in both primary and secondary care in Great Britain (Guo et al. 2016). Decomposition temperatures were obtained for 249 pharmaceuticals (Fig. 1) using the procedure presented in Online Resource 2. A selection of 14 of the most thermally stable pharmaceuticals were selected for use in the waste treatment simulations. Three additional APIs, diclofenac, ethinylestradiol, and carbamazepine, were also selected as these substances have been previously proposed as potential priority substances under the European Water Framework Directive (WFD) along with ibuprofen and estradiol. Ibuprofen and estradiol had already been included in the 14 APIs on the basis of decomposition temperature (Lyons 2014). In the event that these compounds are adopted as WFD priority compounds, better controls of emissions will likely be required in the future. The 17 substances were from a diverse range of therapeutic classes (Table 1).

**Table 1:** The 17 pharmaceuticals selected for testing in the Pyrolysis–gasification waste treatment system (PGWTS) trials, therapeutic class, decomposition temperature, and usage (kg/yr) in Great Britain in 2012

API	Therapeutic class or use class	Decomposition temperature range (°C)	Usage (kg/yr)	Reference decomp. temp
5-Fluorouracil	Anticancer, cytotoxic	282	12,648.7	1
Allopurinol	Antigout	379.5–386	38,593	2
Amantadine	Antiviral/AntiParkinson’s	360	626.6	3
Aspirin	Analgesic	370	96,644.6	4
Atenolol	Beta-blocker	303–335	26,411.5	5
Carbamazepine	Antiepilepsy	190–195	45,331.9	6
Chloramphenicol	Antibiotic, cytostatic	200–704	484.6	7
Diclofenac	Nonsteroidal antiinflammatory drug	>260	16,369.7	8
Estradiol	Hormone	275–317	151.6	9
Ethinylestradiol	Hormone	178	12.9	10
Fluoxetine	SSRI antidepressant	200–300	6200.1	11
Gliclazide	Diabetes	271–429	40,781.2	12
Ibuprofen	Nonsteroidal antiinflammatory drug	180–300	151,739.9	13
Indomethacin	Nonsteroidal antiinflammatory drug	230–330	837.2	14
Ketoprofen	Nonsteroidal antiinflammatory drug	235–400	903.47	15
Sulfamethoxazole	Antibiotic	380–600	1940.2	16
Verapamil	Calcium channel blocker	300–320	6969.9	17

1: Lewis (2007), 2: Samy et al. (2010), 3: RSC (2013), 4: Ribeiro et al. (1996), 5: Pereira et al. (2007), 6: McGregor et al. (2004), 7: Macedo et al. (1999), 8: Tudja et al. (2001), 9: Martin and Wotiz (1962), 10: Cotter et al. (1978), 11: Silva et al. (2007), 12: Zayed et al. (2010), 13: Tita et al. (2011a), 14: Tita et al. (2010), 15: Tita et al. (2011b), 16: Fernandes et al. (1999), 17 Lide and Milne (1994)

### Test Chemicals and Reagents

Allopurinol (≥98 %), amantadine (≥98 %), aspirin (≥99 %), atenolol (≥98 %), carbamazepine (≥98 %), chloramphenicol (≥98 %), diclofenac (≥98 %), estradiol (≥98 %), ethinylestradiol (≥98 %), 5-fluorouracil (≥99 % purity), fluoxetine (≥98 %), gliclazide (≥98 %), ibuprofen (98≥ %), indomethacin (≥99 %), ketoprofen (≥98 %), sulfamethoxazole (≥99 %), and verapamil (≥99 %) were purchased from Sigma-Aldrich (Gillingham, Dorset, UK). Sodium hydroxide solution (50 % in water) and formic acid (≥95 %) used in analytical work were also purchased from Sigma-Aldrich, UK. For the bulk waste trials (see waste treatment simulations), ibuprofen (200 mg), and aspirin (300 mg) tablets were purchased from a local supermarket (Tesco, Cheshunt, UK). Chloramphenicol eye ointment was purchased from Lloyd’s Pharmacy (4 g, 1 % w/w, Martindale Pharmaceuticals, Romford, UK). All solvents used were high performance liquid chromatography (HPLC) grade, methanol (>99.9 %), acetonitrile (>99.9 %), acetone (>99.9 %), and water were purchased from Fisher Scientific (Loughborough, UK).

### Waste Treatment Simulations

The PGWTS trial was designed in accordance with Annex 1, Sect. 1.8 of the Environment Agency Sector Guide EPR5.07 Clinical Waste (Environment Agency (EA) 2011a). The trial was delivered in two phases: Phase 1 included all 17 pharmaceuticals and three waste types. Phase 2, was conducted because results from Phase 1 were inconclusive or indicated that some cross-contamination with select APIs had occurred. Phase 2 included six of the APIs (5-fluorouracil, ibuprofen, ketoprofen, atenolol, estradiol, and ethinylestradiol) and one waste type. All trials were performed at the Pyropure factory in Bordon, Hampshire, UK.

#### Phase 1 Trials

The Phase 1 trials simulated the treatability of the study pharmaceuticals in three waste types: (1) pharmacy take-back waste (termed ‘bulk’ waste) which included blister packs, unused tablets, and packaging; (2) manufacturing production line waste (‘manufacturing’) which included powdered pharmaceutical, placebo tablets, paper towels, overalls, and lab gloves, and (3) healthcare waste which included waste found in a yellow bins such as needles, syringes, packaging, blister packs, and placebo tablets. Details of the composition of each waste simulation are provided in Online Resource 3. For the manufacturing and healthcare waste streams a mixture of the 17 pharmaceuticals, containing between 43 and 430 mg of each API, was added to the waste in a sealed 50 mL polypropylene centrifuge tube. The centrifuge tube was inserted into the middle of the waste load where the heat presumably penetrates last. For the bulk waste, three pharmaceuticals were investigated, aspirin, ibuprofen, and chloramphenicol, and these were added in either tablet or gel form (see Online Resource 4).

For each simulation, the waste matrix and APIs were placed in the PGWTS unit and treated following the manufacturer’s guidelines. There were five runs for each of the three waste streams: two control runs containing just the waste mix (i.e., no pharmaceuticals) and three pharmaceutical runs containing the waste and the pharmaceuticals. The gaseous emission was passed through a water ‘trap’ to collect parent API or their transformation products emitted in the gaseous phase. For each run, three types of samples were collected: liquid effluent, gas trap, and residual solids. Tap water was also taken from the site for analysis. Samples were placed into wide-necked solvent rinsed 125 mL amber glass bottles and stored at −20 °C prior to transportation to the laboratory (at 5 °C) for analysis for APIs.

#### Phase 2

In Phase 2, a mixture of the three wastes, used in Phase 1, was investigated (See Table S1 in Online Resource 3). Ibuprofen was again added in tablet form, the other five APIs were added in a 50 mL centrifuge tube but in these simulations, Vernagel (C_3_H_3_NaO_2_)n was also added to create an insulating layer to surround the powdered APIs. The addition of Vernagel is likely to mimic the effects of excipients that would be present in a real situation. The simulations and sampling followed the same approach as in Phase 1 but additionally, samples from the ashpot, which essentially contains the majority of particles removed from the gaseous stream, were also taken and analyzed (Online Resource 5).

### Preparation of Samples for Analysis for Pharmaceuticals

Each sample was analyzed in triplicate for APIs. The liquid effluent and ‘gas trap’ samples (2 mL in Phase 1 and 3 mL in Phase 2) were concentrated by a factor of ten in Phase 1 and a factor of 60 in Phase 2 using a centrifugal concentrator. Samples were placed inside the centrifugal concentrator overnight until dry, before reconstituting to 200 µL firstly by adding 100 µL of methanol and then by adding 100 µL of HPLC grade water. Samples were vortex mixed after the addition of both the methanol and the water for 10 s each time. The reconstituted sample was then passed through a syringe filter (0.2 µm PTFE (Oasis)) into an amber glass HPLC vial containing a glass insert. In Phase 2, the filtered sample was then concentrated again inside glass insert (contained in the vial) using the centrifugal concentrator. The sample was then reconstituted to 50 µL with 50:50 methanol:water prior to analysis.

APIs were extracted from the sludge using a method adapted from Martin et al. (2010). Briefly, for each replicate 1 ± 0.05 g of sludge (wet weight) was weighed directly into a Pyrex glass tube. The sludge was extracted using ultrasonication with methanol three times (2, 0.5 and 0.5 mL). Samples were ultrasonicated with the Pyrex tubes standing in a beaker filled with deionised water, to the level of the sample liquid, for 15 min each time. After each extraction, samples were centrifuged for 5 min at 960×*g* and the supernatant was combined in a separate glass tube. The supernatant was then dried in the centrifugal concentrator, as per the effluent and gas samples, before reconstituting to 500 µL (Phase 1) with half the volume first added as methanol and then the remainder added as water. The sample was vortex mixed after each addition of solvent. In Phase 2 the total volume was 250 µL. Once reconstituted, samples were passed through a 0.2 µm (13 mm) syringe filter into an amber glass HPLC vial (see note in Online Resource 6 about cleaning of glassware).

For each matrix, the extraction procedure was validated by spiking in a stock solution containing the 17 pharmaceuticals at known concentrations into the appropriate matrix collected from the control runs at seven different levels in the range 0–100,000 ng/mL. Where the calibration series was reasonably linear (R^2^ > 0.9) (Online Resource 7), then the matrix-specific calibration series was used to determine percentage recovery and quantify levels remaining in the sample (see “Results” section). Where the calibration series was not reasonably linear or recoveries were low, the high end standards were compared with solvent standards to determine percentage recovery. In these cases, the calibration was done using the solvent standards and an adjustment made for the percentage recovery.

### Analysis using Liquid Chromatography Triple Quadrupole Mass Spectrometry (LC–MS/MS) and LC - Fourier Transform - Ion Cyclotron Resonance - Mass Spectrometry (LC-FT-ICR-MS)

Concentrations of pharmaceuticals in concentrated samples or sample extracts were determined using an Applied Biosystems/MDS Sciex API 3000 triple quadrupole mass spectrometer interfaced with a Dionex UltiMate^®^ 3000 LCi system, for LC–MS/MS analyses. Positive ion mode was used in Phase 1 and both positive and negative ion modes were used in Phase 2 analyses. For the liquid chromatography, a Dionex Acclaim^®^ RSLC C18 Polar Advantage II column (2.2 µm, 120 Å, 2.1 × 100 mm) was used. Full details of LC–MS/MS methods can be found in Online Resource 8.

The presence of 12 known active transformation products of the 17 parent APIs (not all parent APIs had known active transformation products and some had more than one) in the samples of the emissions from the Pyropure system was assessed. Table S8 (Online Resource 9) presents full details of molecular formulae and monoisotopic mass for the following 12 metabolites: 5-fluoro-2-deoxyuridine 5′-monophosphate, oxypurinol, salicylic acid, carbamzepine-10, 11 epoxide, 4′hydroxy-diclofenac, estrone, estriol, 2-methoxyestradiol, norfluoxetine, 2-hydroxyibuprofen, carboxyibuprofen, norverapamil. We focussed on the transformation to products that would retain pharmacologic activity as this was the primary concern (relating to transformation) for the regulator in England (Personal communication, Robert McIntyre of the Environment Agency, May 2014).

Our aim here was to show that the parent compound was not being broken down into something that also possessed pharmacological activity. We also investigated the mass spectra of any significant chromatographic peak to assess whether large quantities of unknown breakdown products were being produced consistently across samples. Liquid chromatography coupled with an ion cyclotron resonance Fourier transform mass spectrometer (ICR-FT-MS) was used to assess levels of these active transformation products. An Agilent 1200 HPLC was interfaced with a solariX XR 9.4 T (Bruker) FT-ICR mass spectrometer (See Online Resource 9). Where an active metabolite was identified as being the most likely explanation for the signal, semiquantification was made by expressing the peak height of the active metabolite relative to the size of the parent API peak in a standard.

### Calculation of the Percentage Decomposition

Using the total volume of the matrix (effluent, sludge, and water used to collect the gas sample), it was possible to determine the percentage of each API destroyed by relating the concentration in the extracted samples and the starting mass of the API (see Online Resource 10 Tables S9–12). Where the analyses detected nothing, the concentration in the sample was assumed to be half of the limit of detection (LOD) and the percentage of the mass balance that this constituted was calculated accordingly. A value of >99 % decomposition of an API was considered appropriate as the treatment success measure.

### Air Emissions: Other Pollutants

A standard suite of air emissions tests was conducted by EmCO Air Emissions Ltd (Hook, Hampshire, UK), who are a UKAS and M-Certs accredited contractor for testing for particulate matter (PM_10_), nitrogen dioxide, hydrogen chloride, hydrogen fluoride, sulfur dioxide, volatile organic compounds, dioxins, furans, and specific metals (mercury, cadmium, thallium, arsenic, cobalt, chromium, copper, manganese, nickel, lead, antimony, vanadium). Concentrations of these determinands in air emission samples were expressed as a process contribution for the PGWTS using dispersion factors. The percentage that the PGWTS contributed to the environmental assessment limit (EAL) following the H1 guidelines of the Environment Agency (England) for each of the determinands was used to assess whether air emissions from treating pharmaceutical waste would pose a risk to human health or the environment, see Online Resource 11 Table S13 (Environment Agency (EA) 2014).

## Results

The limits of detection of the analytical methods in Phase 1 and Phase 2 are presented in Tables 2 and 3. The percentage recovery of the extraction of pharmaceuticals from the solid material ‘sludge’ is also presented in Table 2.

**Table 2:** Limits of detection (ng/mL) (LOD) for each of the 17 APIs in Phase 1 simulation

API	Recovery of analytical extraction method (%)	LOD (ng/mL)
5-Fluorouracil	28.5	100
Allopurinol	21.4	10
Amantadine	52.1	50
Aspirin	0	100
Atenolol	58.3	100
Carbamazepine	86.8	10
Chloramphenicol	75.2	50
Diclofenac	79.4	10
Estradiol	79.7	500
Ethinylestradiol	79.8	100
Fluoxetine	80.6	50
Gliclazide	45.8	100
Ibuprofen	41.6	10
Indomethacin	13.9	10
Ketoprofen	68.5	50
Sulfamethoxazole	55.5	100

Aspirin could not be recovered from the solids

**Table 3:** Limits of detection (LOD) and quantification (ng/mL) (LOQ) for each of the six retested APIs in Phase 2 simulation in liquid effluent, sludge, ashpot solids, and the air emission

API	Liquid effluent		Sludge		Ashpot		Air	
	LOD (ng/mL)	LOQ (ng/mL)	LOD (ng/mL)	LOQ (ng/mL)	LOD (ng/mL)	LOQ (ng/mL)	LOD (ng/mL)	LOQ (ng/mL)
5-Fluorouracil	35.3	117.6	188.2	627.3	188.2	627.3	313.6	1045.5
Atenolol	2.6	8.6	1.0	3.4	0.9	2.9	1.7	5.7
Estradiol	12.6	42.1	7.8	26.1	15.7	52.2	18.3	60.9
Ethinylestradiol	63.2	210.5	35.5	117.6	70.6	235.3	84.7	282.4
Ibuprofen	180.0	600.0	120.0	400.0	150.0	500.0	300.0	1000.0
Ketoprofen	540.0	1800.0	144.0	480.0	160.0	533.3	32.0	106.7

**Fig. 2:**
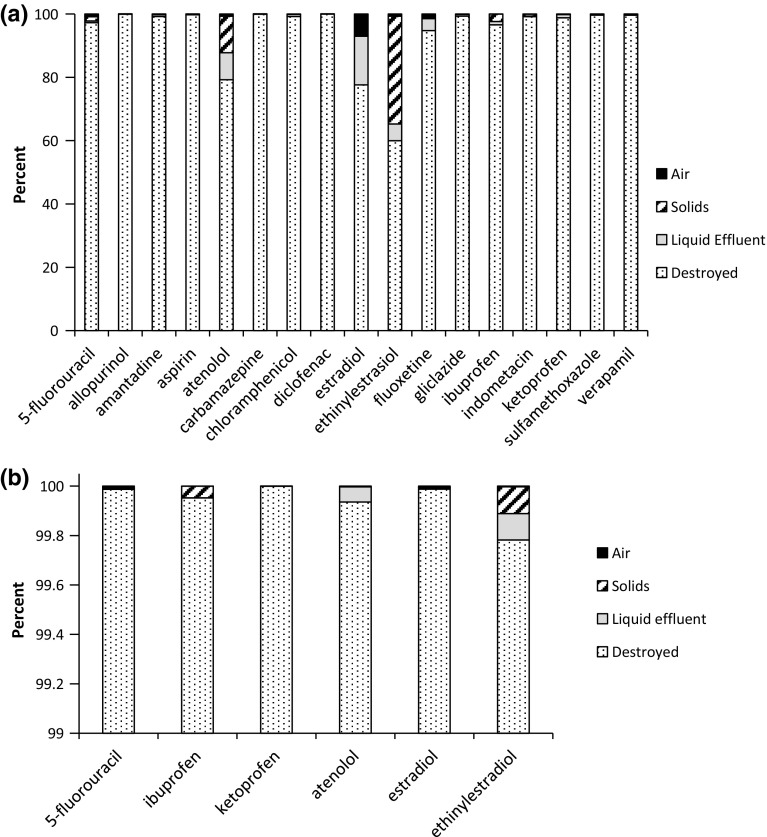
Mass balance showing the fate of pharmaceuticals tested in a Pyrolysis–gasification waste treatment system (PGWTS) in Phase 1 (**a**) and Phase 2 (**b**). The percentage that was destroyed is given by *white-dotted bars*, the percentage found in liquid effluent is given by *gray bars*, in the solids (sludge and ashpot) by diagonally dashed bars and that found in the air trap is given by *black bars*. Note the different y-axis ranges in **a** 0–100 % and **b** 99–100 %

In Phase 1, the PGWTS achieved over 99 % parent API decomposition for ten of the 17 pharmaceuticals (Fig. 2a). The other seven pharmaceuticals had an average destruction of 94 %, with all but three pharmaceuticals having a level of destruction in excess of 90 %, the exceptions being atenolol, estradiol, and ethinylestradiol. There was no apparent effect of waste type on the ability of the PGWTS to destroy pharmaceuticals. The frequency of detection was highly variable between matrices and pharmaceuticals (see Online Resource 12, Tables S14 and S15 for a full breakdown of the frequency of detection). In Phase 1, five pharmaceuticals were detected in the controls (corresponding to 0.01–0.23 % of the added APIs) and 11 in tap water (corresponding to 0.01–16.1 % of added APIs, mean = 2.3 %) (Online Resource 12 and 13). The contamination of tap water is believed to result from cross-contamination during the sampling, as additional checks of the factory’s tap water taken a month after the trials, and sump and chamber drain water taken from the same machine used in the trials did not show any trace of the seventeen pharmaceuticals. This highlights one of the challenges in performing studies of this type at an industrial site (such as the Pyropure facility) when using highly sensitive analytical equipment. Due to the presumed cross-contamination, a second trial was performed on those active ingredients where destruction levels corresponding to <99 % destruction were determined in the Phase 1 simulation samples. In these Phase 2 trials, the destruction levels for the worst performing APIs from Phase 1 were above 99.9 % for five APIs and above 99.7 % for the other API (ethinylestradiol) (Fig. 2b).

Three active metabolites were detected in Phase 1 and five in Phase 2 samples (out of 12 metabolites searched for). Where they were detected, with the exception of 5-fluoro-2-deoxyuridine 5′-monophosphate (F-dUMP), the semi-quantification suggests that the levels are likely to be such that they are of less concern than their parent compounds (Table 4). In Phase 1, the amount of the active metabolite of 5-fluorouracil, F-dUMP, in air was approximately 25.2 % of the added amount of 5-fluorouracil, 60.3 % in liquid effluent but less than 0.001 % in the sludge. The amount of F-dUMP may well be overestimated due to differences in sensitivity for the metabolite and parent compound. The presence of active metabolites in control samples, of parent compounds that were not included in these runs, suggests that cross-contamination occurred in Phase 1 simulations. In Phase 2, the highest (approximate) percentage of added 5-fluorouracil converted to F-dUMP was only 0.54 % (in liquid effluent). F-dUMP could not be detected in air and was less than 0.1 % of added 5-fluorouracil in the ashpot and sludge. Significant quantities of unknown transformation products were not consistently identified across samples in the mass spectra.

**Table 4:** Active metabolites detected in Phase 1 and Phase 2, in samples of air, mains water (i.e., water straight from the tap taken at the same time as the unit was being drained and washed out with tap water), liquid effluent, the solid ashpot residue and the sludge (solid part of the effluent)

	Phase 1		Phase 2					
			API run 1		API run 2		API run 3	
	Metabolite	Approximate parent equivalent %	Metabolite	Approximate parent equivalent %	Metabolite	Approximate parent equivalent %	Metabolite	Approximate parent equivalent %
Air	F-dUMP	25.2	–	–	–	–	2-me	0.03
Mains water	NA	NA	2-hydoxyIBF 2-me	0.028 0.050	2-me	0.010	2-me	0.007
Liquid Effluent	F-dUMP 2-me SA	60.3 0.04 0.35	–	–	F-dUMP	0.54	2-hydroxyIBF	0.014
Solid Ashpot	NA	NA	2-me F-dUMP Est	0.00002 0.02 0.00003	2-me F-dUMP Est	0.00007 0.012 0.0003	carboxyIBF Est	0.0005 0.0005
Sludge rep 1	2-me	0.0007	2-me 2-hydroxyIBF F-dUMP carboxyIBF Est	0.007 0.012 0.024 0.006 0.0016	2-me 2-hydroxyIBF carboxyIBF Est	0.007 0.00011 0.0040 0.00014	2-me F-dUMP Est	0.00009 0.042 0.00014
Sludge rep 2	NA	NA	Est	0.0015	2-me 2-hydroxyIBF carboxyIBF Est	0.00009 0.011 0.0056 0.000023	2-me F-dUMP carboxyIBF Est	0.0001 0.043 0.001 0.0007

In Phase 1, means are presented for the three waste types (bulk, manufacturing, and sharps as only 15 samples were run in total (3 air, 3 sludge, and 9 liquid effluent). In Phase 2, a larger number of samples were analyzed and so the data are presented separately for each run. The metabolites detected were: 5-fluoro-2-deoxyuridine 5′-monophosphate (F-dUMP)), 2-methoxyestradiol (2-me), estrone (Est), 2-hyroxyibuprofen (2-hydroxyIBF), and carboxyibuprofen (carbIBF). Where an active metabolite was detected, the concentration was estimated in terms of parent equivalent and then related to the percentage of the starting mass that this was equivalent to. For control and blank runs containing only Polyethylene terephthalate (PET) and all mains water samples, no API was added and so detection must be due to background levels in the tap water. Note the F-dUMP parent equivalent is likely to be overestimated due to the low sensitivity of the MS assay for its parent compound

None of the non-API determinands monitored for in the air emission studies was found to exceed short- or long-term EALs. The highest process contribution for any determinand was for PM_10_ (0.41 %). Full data on the process contributions for each determinand can be found in Online Resource 11.

## Discussion

This study was conducted to provide the Environment Agency with evidence of the efficacy of an alternative (to high-temperature incineration) treatment technology for treating pharmaceutically contaminated waste (EA 2011b). We are not aware that others have done this before, and if they have, this information would be commercially sensitive, so it is unlikely we would gain access. Indeed, we are not aware of any studies testing the efficacy of high-temperature incineration, or any thermally or chemically (e.g., alkaline hydrolysis) based alternative treatment technology for pharmaceutically contaminated wastes (World Health Organization (WHO) 2014). This makes our data timely, novel, and relevant to the needs of the healthcare sector, regulators, and the waste management and pharmaceutical industries.

The results indicate that in situ PGWTS offer an effective treatment process for pharmaceutically contaminated wastes. When the findings of both trials are combined, all parent APIs were demonstrated to be more than 99 % decomposed by the PGWTS (or below the LOD as with fluoxetine). Analysis for known transformation products in Phase 1 suggested that the formation of known active metabolites and breakdown products was negligible except for F-dUMP. F-dUMP acts slower than its parent compound but is known to be more toxic to cells (Matuo et al. 2009). The fact that in excess of 85 % of the added 5-fluorouracil (in Phase 1) was detected as F-dUMP, when expressed as parent equivalents, was potentially a concern. Thus in Phase 2, we increased the amount of 5-fluorouracil added to the PGWTS. This revealed a maximum of only 0.5 % of the added 5-fluorouracil which was detected as F-dUMP. It is probable that the detection of F-dUMP in Phase 1 was an issue of contamination which was overestimated due to the low sensitivity for the parent compound.

Based on the evidence of Phase 1 and 2, it is unlikely that levels of active metabolites and transformation products discharged from the PGWTS would be of concern to human health and the environment. Nevertheless, further research is needed, particularly in the area of transformation products. The gaseous emission from the PGWTS was sampled by bubbling through water. Therefore, only water-soluble APIs and transformation products would be collected. All 17 parent APIs and known active metabolites are sufficiently water soluble for this method to enable their collection should they have been present (e.g., see www.drugbank.ca). While the insoluble component of the effluent is essentially the solids that we extracted with a polar solvent (Martin et al. 2010), we cannot rule out nonpolar transformation products, originating from either the APIs (Kern et al. 2010) and/or other components of the simulated waste mix. For example, these could include water-soluble organics such as alcohols, ethers, aldehydes, and carboxylic acids; pyrolytic oils e.g., (asphaltenes, aliphatics, aromatics, or polars), gases containing carboxyl groups; and pyrolytic chars with a high ash content (Karyaldirim et al. 2006). Determining whether this is the case is now a priority for research into the efficacy of PGWTS for treating pharmaceutically contaminated wastes.

Nevertheless, our data suggest PGWTS could provide an in situ alternative to high-temperature incineration. By providing households, pharmacies, hospitals, and manufacturers with a convenient and safe alternative to disposing of unused medicines to sewer or in solid waste PGWTS could reduce the amounts of APIs being inappropriately disposed of. Ultimately, PGWTS could contribute to reducing pharmaceutical contamination of both the terrestrial and aquatic environments (e.g., McClellan and Halden 2010; Kasprzyk-Hordern et al. 2010).

Inappropriate disposal of unused pharmaceuticals has been identified as an issue in several developed countries (Daughton and Ruhoy 2009, 2013; Thach et al. 2013; Tong et al. 2011). Data are highly variable among countries, and in some cases among studies conducted within the same country. A number of factors could affect the data reported by these studies: e.g., data collection method, question types, demographics, sample size, and changes in attitudes over time. Studies in Sweden and Germany (Persson et al. 2009; Tong et al. 2011) found only 3 and 7 %, respectively of unused medicines were disposed of in household waste, while Lithuania and Kuwait had 87–89 % and 97 %, respectively (Krupiene and Dvarioniene 2007; Abahussain and Ball 2007; Abahussain et al. 2006). However, in the US (45–54 %; Kotchen et al. 2009; Glassmeyer et al. 2009), the UK (63 %, Bound and Voulvoulis 2005), and Republic of Ireland (51 %, Vellinga et al. 2014) disposal in household waste appears similar. For disposal down the sink or toilet, <1 % did this in Sweden and New Zealand (Persson et al. 2009; Braund et al. 2009) while in the US 54 % dispose of medicines to the toilet and 35 % down the sink (Seehusen and Edwards 2006; Glassmeyer et al. 2009). Other fates for unused medicines included storing at home in case of future personal use or needs of a friend/relative, burning, placing in recycling boxes, or disposing to hazardous waste.

Providing the public with knowledge of appropriate disposal methods for unused medicines and ensuring convenient access to take-back strategies should help to decrease inappropriate disposal (Persson et al. 2009; Thach et al. 2013). In Sweden, as many as 85 % of respondents knew that returning unused medicines to the pharmacy was the appropriate way to handle them, and 43 % said they had done so in the last 12 months (Persson et al. 2009). However, in the UK, a 2013–14 survey suggested only 17 % of people use take-back strategies (Williamson and Boxall 2014, unpublished data) and as many as 30 % of people were found to dispose of medicines inappropriately. Furthermore, a study in the Republic of Ireland found only 6 % used take back and 72 % disposed of medicines inappropriately (Vellinga et al. 2014). However, this is still better than reported participation in the USA (1.4 %) (Glassmeyer et al. 2009). Based on these studies, it appears a lack of knowledge around appropriate disposal and access to take-back strategies are the main reasons for inappropriate disposal (Thach et al. 2013). Furthermore, initiatives that have not advised best practice for handling unused medicines such as the SMARxT disposal partnership in the USA are also likely to have contributed to pharmaceutical emissions from landfill. This initiative recommends that unused medicines are crushed, mixed with unappealing material such as cat litter (to prevent scavenging and illicit use) and disposed of to landfill in a sealable bag or box. Such strategies are unlikely to protect the environment, as a sealed bag or box can easily be crushed and opened in the landfill (Musson and Townsend 2009). The pharmaceuticals can then end up in leachate. Both leachate and flushed (or rinsed) medicines can end up in the WWTP where removal is often incomplete, thus leading to contamination of the aquatic environment (Daughton 2003).

The study of pharmaceutical contamination of leachate has been largely neglected (Musson and Townsend 2009). Recent studies in the US and Spain have detected several pharmaceuticals at the µg/L level in landfill leachate (including amoxicillin, carbamazepine, furosemide, ibuprofen, and omeprazole) (United States Geological Survey (USGS) 2014; Rodríguez-Navas et al. 2013). Lubick (2010) reported details of a similar study from Maine in which paracetamol was detected in leachate at 117 µg/L, ciprofloxacin at 269 ng/L, and even cocaine was detected at 57 ng/L. Many landfills pipe their leachate to wastewater treatment plants (Musson and Townsend 2009). Some healthcare facilities advise staff to flush unused medicines down the toilet or rinse down the sink and in these instances APIs will be released directly to the sewerage system (Daughton 2003; Boxall et al. 2014; Mackridge 2005).

In healthcare facilities where medicines are rinsed down the sink, antibiotics are often used (Bumpass et al. 2014). Antibiotic resistance (Starlander and Melhus 2012), and particularly resistance to ‘last resort’ carbapenem antibiotics (Kotsanas et al. 2013), is of particular concern. Repeated washing of traces of these medicines down can lead to the development of a biofilm containing persistent gram-negative bacteria from multiple genera (Kotsanas et al. 2013). This biofilm acts as a reservoir for the transmission of antimicrobial resistance making nosocomial transmission highly likely (Kotsanas et al. 2013). Cleaning and replacing sinks with better designed ones have both been suggested by some as the way towards antimicrobial stewardship (Kotsanas et al. 2013). However, this is a rather narrow-minded view of stewardship which simply transfers the problem out of the hospital and into the environment. PGWTS and other in situ waste treatment technologies offer a real solution. By destroying unused antibiotics and antibiotic contaminated waste at source, the risks of these drugs, and the transfer of antimicrobial-resistant bacteria to the environment in this way could be eliminated. This could also be important at manufacturing plants. Although manufacturing emissions of pharmaceuticals to the environment are concentrated in specific areas, they may also be significant, particularly for antibiotics, as their emission even at trace levels will promote the development of antimicrobial-resistant microorganisms (Larsson 2014). To this extent, PGWTS could still have a significant role to play in terms of human and environmental health at the global scale (Larsson 2014).

In situ PGWTS could realistically be installed at pharmacies, manufacturers’ sites, hospitals, and healthcare facilities across the world. The adoption of alternative treatment technologies such as PGWTS could make take-back strategies much more effective than they currently are. Pharmacies and clinics are likely to be willing participants in such strategies as in situ waste management comes with economic incentives in addition to environmental benefits. By removing the need to transport waste across potentially long distances to high-temperature incinerators (there are currently only 22 in the whole of the UK (DEFRA 2013)), fuel, labor, and security (in the case of wastes containing controlled substances such as morphine) as well as reducing CO_2_ emissions. The cost-benefits to the user of using PGWTS over separate collections and high-temperature incineration are compelling, which is an important factor in the likelihood of their incorporation in take-back strategies. For a typical pharmaceutically contaminated medical waste, which has a high plastic content and assume its calorific value is 27 MJ/kg, the electrical cost to process this in a PGWTS is approximately $300 per metric tonne. Factoring in costs associated with regulatory requirements, water, and maintenance costs adds an additional $180 per metric ton, giving a total running cost of $480 per metric ton for PGWTS.

A waste producer can pay between $3748 and $4410 per metric ton (or $1.70 to $2.00 per lb) for collection, transport, and treatment of hazardous waste (Rich et al. 2013). Taking the midrange figure of $4000 per metric ton, the on-site PGWTS could save the user around $3520 per ton of waste. In addition to these costs savings, the PyroPure PGWTS generates 2.68 kW h per ton of usable energy in the form of heat for a high plastic content load. This means a user processing 20 tons per year would generate 53,600 kW h of free heat (worth the equivalent of $5400 in replacing electricity at $0.1/kW h). Combining the value of energy generated with reduced costs in waste collection and processing, PGWTS offer approximately 95 % cost savings compared to collection high-temperature incineration. To purchase a single PGWTS currently costs approximately $75,000. For a user treating around 20 metric tons per year, the PGWTS investment would start to make cost savings in a little over a year. These figures suggest PGWTS are economically viable and environmentally friendly which could help improve the density with which take-back strategies are offered to a population.

As it is estimated that up to 65 % of prescribed medicines remain unused by patients (Boxall et al. 2014), but only around 17 % are being safely disposed of in the UK (Williamson and Boxall 2014, unpublished data) (ranging from 1.4–65 % in other Western countries) (Tong et al. 2011; Persson et al. 2009; Glassmeyer et al. 2009; Vellinga et al. 2014; Musson and Townsend 2009; Daughton 2003; Seehusen and Edwards 2006; Musson et al. 2007; Isacson and Olofsson 1999; Cameron 1996), there is clearly a significant proportion of these unused medicines which are disposed of in ways that result in environmental contamination. Any technology that will help to close the gap between what is unused and what is disposed of in take-back strategies can only be beneficial to the environment.

To ensure the success of PGWTS, we believe they should be implemented as part of wider stewardship strategies which involves a wide range of stakeholders. Doctors should prescribe smaller doses to help reduce the number of unused medicines in households (Daughton and Ruhoy 2013). Governments and manufacturers should set up education and advertising strategies to raise the public’s awareness of the availability of take-back facilities in their area while highlighting the consequences of flushing to sewer or disposing of medicines in household waste (EU 2015).

Changing people’s attitudes and raising awareness of take-back strategies will be paramount to a successful outcome. Strategies such as the EU’s ‘no pills in waters’ cooperation project have been in place for a while in Europe to encourage greater use of take-back strategies (EU 2015). In such strategies, partnerships between environmental, social scientists, and communications and software experts have been demonstrated to be very important to gain an idea of the scale of the problem and create a pathway towards making changes for the better. For example, connecting with young people to inform them about the issue of pharmaceuticals in the environment was identified as an important issue to ensure the future sustainability of such strategies (EU 2015). Other means of connecting with demographic groups (e.g., the elderly and their carers) could be through strategies that introduce information boards in doctors’ surgeries, pharmacies, placing adverts on healthcare-related websites and adding labels to pharmaceutical packaging.

## Conclusions

PGWTS have the potential to make significant reductions to current levels of pharmaceutical contamination in the environment. Not only are there potential benefits in terms of pharmaceutical contamination of the environment, there are also financial and environmental incentives. This makes PGWTS a viable alternative to waste collection and transport to high-temperature incinerators. The initial investment in a PGWTS could be repaid in a little over a year due to approximately 95 % cost-saving compared with high-temperature incineration (Rich et al. 2013). Alternative treatment (to high-temperature incineration) technologies for pharmaceutically contaminated waste will only be effective if the public and healthcare workers gain a greater awareness of the consequences of inappropriate disposal of API-containing wastes and with it a sense of environmental responsibility. Education can pave the way towards a cultural change in the way that we deal with unused medicines and in situ waste treatments will represent a convenient disposal option that can help smooth this transition.

## Electronic Supplementary Material

Below is the link to the electronic supplementary material.

Supplementary material 1 (DOCX 347 kb)

## Abbreviations

(PGWTS): Pyrolysis–gasification waste treatment system
