# Supplementary material for: Evaluation of a Novel Approach for Reducing Emissions of Pharmaceuticals to the Environment
Source: Environ Manage. 2016 Jun 24;58(4):707–20. doi: 10.1007/s00267-016-0728-9 (PMC5026718; doi:10.1007/s00267-016-0728-9)
Supplement: Supplementary file 1 — Supplementary material 1 (DOCX 347 kb) [file 267_2016_728_MOESM1_ESM.docx]

Evaluation of a novel approach for reducing emissions of pharmaceuticals to the environment

*Thomas G. Bean* ^1^, Ed Bergstrom ^2^, Jane Thomas-Oates ^2^, Amy Wolff ^3^, Peter Bartl ^3^, Bob Eaton ^3^ and Alistair B.A. Boxall ^1^*

1. Environment Department, University of York, UK YO10 5DD
2. Centre of Excellence in Mass Spectrometry and Department of Chemistry, University of York, UK YO10 5DD
3. Pyropure Ltd. Unit 58 Woolmer Trading Estate, Bordon, Hampshire, UK GU35 9QF

* Author for correspondence: Thomas G. Bean

Current address: Department of Environmental Science and Technology, University of Maryland, USA MD 20742

email: tbean@umd.edu

Telephone: +1 301 497 5713

Fax: +1 301-497-5624

**1. Emissions from the Pyropure system**

The effluent from Pyropure (an example of a Pyrolysis-gasification waste treatment system (PGWTS)) originates from two sources. The sump (gas ‘scrubber’ water) contains a high-pressure pump used to feed a number of fine mist sprays, which are used to cool the exhaust gases, remove particulate matter from the gas stream and absorb water-soluble gases. The sump water is dispelled throughout the cycle as the pH or temperature reaches its set point. The chamber drain water originates from flushing/cleaning the main waste-processing chamber at the end of each cycle and contains the solid fraction of the waste. During the trials, all of the sump and chamber drain water were combined before being sampled. The remaining effluent was then pumped to a bunded holding tank for safe storage until the end of the trial period to ensure appropriate disposal. Under normal operating conditions, both sump and chamber drain water are discharged directly to the sewer.

**2. Selection of test pharmaceuticals**

Pyropure normally operates at a minimum temperature of 550 ^o^C. Starting with usage data, decomposition temperatures for the 300 most used APIs in both primary and secondary care were searched for in databases such as Toxnet (US Environmental Protection Agency), Drugbank and the Web of Science. Typical search terms included:

- pharmaceutical name
- decomposition temperature
- differential scanning calorimetry (DSC)
- thermal gravimetric analysis (TGA)
- thermal stability.

Frequently, the drug’s decomposition temperature was either unavailable or not clearly reported in papers or databases. In these cases, the team applied the approach presented in Figure S1 to try and obtain the temperatures, using one of two main techniques of thermal analysis:

1) **Differential scanning calorimetry (DSC)** – which looks at heat transfers that do not involve changes in mass. Melting and vaporisation are endothermic events while decomposition is exothermic. Therefore if an endothermic peak is followed by an exothermic peak, the decomposition temperature could be deduced

2) **Thermogravimetric analysis (TGA)** – a mass change of a substance as a function of temperature. The substance is subjected to a controlled temperature programme which helps identify decomposition temperature as the mass drops significantly and rapidly as volatile decomposition products are released.


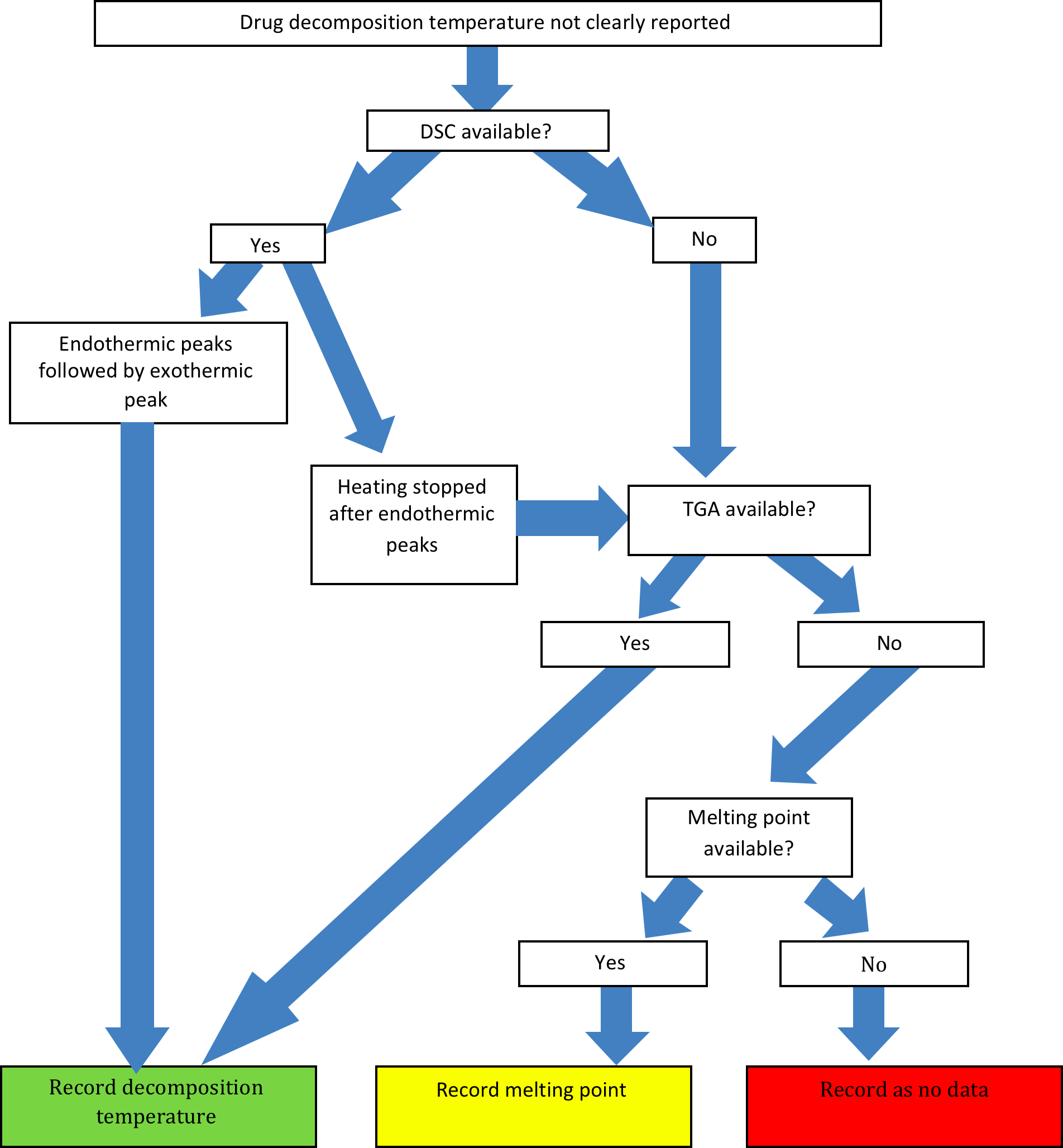
In addition, we contacted a number of leading pharmaceutical manufacturers (Roche, Johnson & Johnson, AstraZeneca, Novartis and Pfizer) for assistance with the literature review. However, the manufacturers tended only to have data on melting points, and not on decomposition temperatures, as melting point information can be used to determine the purity of a substance.

**Figure S1:** The process used to identify decomposition temperatures of pharmaceuticals in cases where the decomposition temperature was not clearly reported.

**3. Waste types used in the trials**

**Phase 1**

In phase 1, the three waste types were designed to simulate:

1: Take-back waste typical of that received by a pharmacy or bulk waste found at a manufacturing plant (referred to as ‘bulk waste’). This waste stream usually contains a mixture of tablets in their original packaging which could be a blister pack inside a cardboard or a plastic container. The simulated mix was: a 60-litre cardboard box; 250 g of placebo tablets; 400 g of blister packs; and 300 g of plastic coated cardboard boxes.

2: Production line waste from a manufacturing plant (referred to as ‘manufacturing waste’). Manufacturing waste is likely to include small amounts of pharmaceutical spilled or cleaned up from the production line e.g. on overalls, gloves and lab roll. To simulate this waste stream accurately, samples of uncontaminated production line waste was obtained from a pharmaceutical manufacturer (Actavis, Devon, UK). The sample contained: 1.5 kg disposable overalls; 1.6 kg blue paper towels; 1.5 kg packaging (including blister pack material and plastic coated cardboard packaging); and 0.05 kg disposable gloves.

3: Yellow bin healthcare waste (referred to as ‘sharps waste’). This waste stream was designed to simulate the yellow bins collected from healthcare facilities which could include pharmaceutical residues on used needles/syringe bodies or unused product disposed of directly to the yellow bin. This simulated waste mix contained: six lots of 5 L sharps bins, the total weight per run being 1.45 kg, with a mixture of simulated soft clinical wastes and sharps (with syringe bodies containing benign liquids) inside.

**Phase 2 waste mix**

When the experiment was repeated in phase 2, a general waste to simulate clinical waste was used as this was considered to be the worst case scenario. All three wastes types were not retested because in phase 1, the waste type did not appear to impact the overall result. The phase 2 waste type selected is summarised in Table S1.

**Table S1**: The composition and masses (g) of each component of the general waste mix used in the phase 2 trials.

|  | **Weight each (g)** | **Quantity** | **Total**  **(g)** |
| --- | --- | --- | --- |
| Overalls | 186 | 2 | 372 |
| Blue paper | 100 | 1 | 100 |
| Hand towels | 2.4 | 30 | 72 |
| Blister packs | 250 | 1 | 250 |
| Gloves | 8.3 | 6 | 49.8 |
| Card | 450 | 1 | 450 |
| Placebos | 250 | 250 | 250 |
| Total | - | - | 1,543.8 |

**4. Masses of API used in the trials**

During the ‘bulk’ pharmaceutical run in phase 1, the pharmaceutical mixture was not introduced in powder form, but instead we purchased and used available over-the-counter medicines. In the bulk runs, the total amounts of APIs introduced into each run were as follows: aspirin (tablet) 3.6 g; ibuprofen (tablet) 2.4 g; and chloramphenicol (liquid) 0.04 g. Unfortunately, only these three APIs were available for purchase over-the -counter and it was not possible to obtain more tablets for the trial in the time available. These tablets/ liquids were also inserted into the middle of the waste load.

Table S2 contains the masses of API used in powder form in the trials

**Table S2:** Masses of pharmaceuticals (mg) in powder form added to manufacturing waste and soft/ clinical sharps waste in phase 1. In phase 2, all pharmaceuticals except ibuprofen were added in powder form to a general waste mix, ibuprofen was added in tablet form (10 × 200 mg) hence no standard error in mass could be calculated.

|  | **Phase 1** | | **Phase 2** | |
| --- | --- | --- | --- | --- |
| **API** | **Mean Mass**  **(mg)** | **Standard Error (mg)** | **Mean Mass**  **(mg)** | **Standard Error (mg)** |
| 5-Fluorouracil | 215.8 | 0.074 | 1070.7 | 3.4 |
| Allopurinol | 215.9 | 0.074 | - | - |
| Amantadine | 429.5 | 0.15 | - | - |
| Aspirin | 430.0 | 0.15 | - | - |
| Atenolol | 43.4 | 0.015 | 1121.5 | 38.8 |
| Carbamazepine | 221.1 | 0.076 | - | - |
| Chloramphenicol | 428.9 | 0.15 | - | - |
| Diclofenac | 430.0 | 0.15 | - | - |
| Estradiol | 42.8 | 0.015 | 1624.6 | 37.9 |
| Ethinylestradiol | 43.0 | 0.015 | 1016.3 | 9.4 |
| Fluoxetine | 14.7 | 0.0051 | - | - |
| Gliclazide | 213.9 | 0.074 | - | - |
| Ibuprofen | 42.8 | 0.015 | 2000 | NA |
| Indomethacin | 215.0 | 0.074 | - | - |
| Ketoprofen | 214.1 | 0.074 | 1033.4 | 18.5 |
| Sulfamethoxazole | 428.3 | 0.15 | - | - |
| Verapamil | 43.0 | 0.015 | - | - |

**5. Sampling of waste from the PGWTS**

- **The liquid effluent** **samples** were a pro-rata combination of the sump and chamber drain water (see note above on emissions from the PGWTS) after removal of the settleable solids (see solid material, below). In triplicate, 100 mL samples were collected using a measuring cylinder.
- **Air or gas emission samples** were collected by bubbling the air emission through a jar of water containing exactly 600 mL of tap water. All of the selected pharmaceuticals were water soluble so would dissolve in the water if any of them remained in the gaseous emission. A single 100 mL sample was collected from the well mixed 600 mL in the jar at the end of each run.
- **Solid material or sludge samples** were extracted from the effluent at the end of each run, by leaving the combined chamber and sump water from each run to settle for 3-4 hours in a bucket. Normally, PGWTS discharges all effluent to sewers and the solids are contained within this discharge. However, as the dry solids could contain the majority of the non-dissolved contaminants, we analysed these as a sample in their own right. In order to extract the dry solids from the effluent, the wet settleable solids, referred to hereafter as ‘sludge’, were collected. As the solids settled to the bottom, the water was gradually decanted from the surface using a 5 L bucket and discarded it to an on-site bunded tank, to ensure appropriate disposal of all residues at the end of the trial. This process was repeated until the solids contained as little water as possible, enabling transfer to successively smaller containers, until all of the solid-in-water suspension could be transferred to 4-5 of the 125 mL sample bottles for transportation to the University of York. In the laboratory, more water was removed prior to extracting the solids by passing the samples through pre-rinsed (deionised water) Whatman no.1 filter paper (Fisher Scientific, Loughborough, UK). The sludge was extracted on a wet weight basis with triplicate samples taken to determine the percentage dry mass.

**Sampling the ashpot in Phase 2**

In the PGWTS, emissions leaving the main chamber are passed through a cyclone, before being cleaned and converted in the catalyst. These particles from the cyclone are collected in an ‘ashpot’, before being rinsed and sent to sewer at the end of each cycle. In phase 1 trials, the ashpot contents was simply merged with the sludge from the chamber and sampled. By keeping the ashpot and sludge from the chamber separate we could understand whether the pharmaceuticals were becoming airborne and going through the catalyst into the ashpot.

**Appendix 6. Quality control and quality assurance**

***Standard operating procedure for cleaning laboratory glassware***

## For each sample, new equipment was used and disposed of with the exception of the Pyrex glass test tubes. Between samples, these tubes went through a rigorous standardised cleaning process which involved manual scrubbing with detergent, 10 rinses with tap water, 2 hours in an ultra-sonication bath with detergent, 10 rinses with tap water, 1 hour in an ultrasonication bath in 5 M nitric acid, 10 rinses with tap water, a deionised water rinse, an acetone rinse, a methanol rinse and finally an HPLC water rinse. Tubes were left in a drying cabinet overnight before reusing.

**Appendix 7. Calibration series**

The R^2^ values for the LC-MS/MS calibration series used in phase 1 and phase 2 are presented in Table S3.

**Table S3:** R^2^ values for calibration series in phase 1 and phase 2 experiments. In phase 1, standards made up in 10:90 methanol:water (solvent standards) were used for all calibrations due to cross contamination which skewed the standards at the lowest concentrations and so percentage recovery and or matrix effects had to be accounted for using the standards at the highest concentrations (10,000, 50,000 and 100,000 ng/mL). For the air and liquid effluent standards, the sample matrix matched the solvent standards closely and so no adjustment for matrix effects was made.

| **Parent compound** | **Phase 1** | **Phase 2** | | | |
| --- | --- | --- | --- | --- | --- |
|  | **Solvent** | **Air** | **Water** | **Sludge** | **Solvent** |
| 5-Fluorouracil | 0.98 | 0.98 | 0.98 | 0.83 | 0.99 |
| Allopurinol | 0.99 |  |  |  |  |
| Amantadine | 0.98 |  |  |  |  |
| Aspirin | 0.99 |  |  |  |  |
| Atenolol | 0.99 | 0.97 | 0.98 | 0.99 | 0.99 |
| Carbamazepine | 0.99 |  |  |  |  |
| Chloramphenicol | 0.99 |  |  |  |  |
| Diclofenac | 0.99 |  |  |  |  |
| Estradiol | 0.97 | 0.82 | 0.86 | - | 0.99 |
| Ethinylestradiol | 0.97 | - | - | - | 0.99 |
| Fluoxetine | 0.99 |  |  |  |  |
| Ibuprofen | 0.94 | 0.98 | 0.98 | 0.89 | 0.99 |
| Indometacin | 0.99 |  |  |  |  |
| Gliclazide | 0.99 |  |  |  |  |
| Ketoprofen | 0.99 | 0.89 | 0.91 | 0.93 | 0.97 |
| Sulfamethoxazole | 0.99 |  |  |  |  |
| Verapamil | 0.93 |  |  |  |  |

**8. Analysis using LC-MS/MS**

Phase 1

Multiple reaction monitoring (MRM) transitions for the 17 pharmaceuticals are given in Table S4. For the liquid chromatography, a multistep gradient with solvents A: H_2_O 0.1% formic acid, B: acetonitrile 0.1% formic acid, was used at a flow rate of 200 µL min^-1^ with a total run time of 23 minutes. The gradient was as follows: one minute 10% B; a ramp increase to 80% B at 18 minutes; a step increase at 19.1 minutes to 95% B, held for two minutes; conditions returned to 10% B to equilibrate until the end of the run. To optimise the sensitivity of the measurement, the 23-minute run time was divided into five periods, so that the mass spectrometer was recording a maximum of five analytes during any one period. The retention time and periods into which each of the 17 analytes fell are given in Table S5.

Phase 2

In phase 2 six pharmaceuticals were re-tested. Negative ion mode was used for 5-fluorouracil, ketoprofen and ibuprofen and positive ion mode for atenolol, estradiol and ethinylestradiol. Multiple reaction monitoring (MRM) transitions for both positive and negative ion mode are given in Table S6.

A multistep gradient with solvents A: H_2_O 0.1% formic acid, B: acetonitrile 0.1% formic acid, was used at a flow rate of 200 µL min^-1^ with a total run time of 11.5 minutes for both positive and negative ion mode analyses. The gradient was as follows: zero minutes 5% B; a gradient increase to reach 65% B at 0.2 minutes; a gradient increase to reach 83% B at 6 minutes, a step increase to 95% B at 6.1 minutes, 95% B was held for 1.9 minutes before conditions were returned to 5% B at 8.2 minutes to equilibrate until the end of the run.

For negative ion mode, high pH was used to aid deprotonation using post column addition of a solution of 2.5% aqueous ammonium hydroxide (Sigma Aldrich, UK) (so not to interfere with the retention times on the column) using the loading pump at a rate of 10 µL/min.

To optimise the sensitivity of the measurement, the 11.5-minute run time for both positive and negative ion mode was divided into two periods, so that the mass spectrometer was recording a maximum of two analytes during any one period (see Table S7).

**Table S4:** Phase 1 MRM transitions and optimised instrumental settings DP=De-clustering Potential, FP=Focussing Potential, EP= Entrance Potential, CE=Collision ‘Energy’ setting, CXP= Collision Cell Exit Potential.

| **API** | **Product ion (*m/z*)** | **Precursor ion (*m/z*)** | **DP**  **(V)** | **FP**  **(V)** | **EP**  **(V)** | **‘CE’ setting**  **(V)** | **CXP**  **(V)** |
| --- | --- | --- | --- | --- | --- | --- | --- |
| 5-Fluorouracil | 131.1 | 114.0 | 30 | 100 | 15 | 22 | 18 |
| Allopurinol | 137.1 | 110.3 | 50 | 320 | 14 | 30 | 20 |
| Amantadine | 152.2 | 107.2 | 34 | 245 | 13 | 36 | 21 |
| Aspirin | 181.0 | 121.1 | 8 | 150 | 10 | 8 | 20 |
| Atenolol | 266.4 | 225.1 | 19 | 200 | 10 | 23 | 18 |
| Carbamazepine | 237.1 | 194.3 | 20 | 200 | 10 | 30 | 15 |
| Chloramphenicol | 323.2 | 275.1 | 40 | 150 | 10 | 20 | 15 |
| Diclofenac | 296.3 | 215.2 | 28 | 200 | 9 | 28 | 18 |
| Estradiol | 273.3 | 255.2 | 32 | 200 | 12 | 15 | 15 |
| Ethinylestradiol | 297.4 | 279.4 | 30 | 200 | 11 | 17 | 8 |
| Fluoxetine | 310.2 | 147.9 | 26 | 175 | 10 | 13 | 15 |
| Gliclazide | 323.7 | 127.1 | 60 | 200 | 14 | 28 | 10 |
| Ibuprofen | 207.0 | 161.4 | 22 | 200 | 12 | 15 | 15 |
| Indomethacin | 358.1 | 139.1 | 60 | 200 | 10 | 24 | 11 |
| Ketoprofen | 255.1 | 209.2 | 80 | 200 | 9 | 20 | 18 |
| Sulfamethoxazole | 253.5 | 156.0 | 42 | 300 | 15 | 22 | 11 |
| Verapamil | 455.0 | 303.3 | 60 | 320 | 14 | 30 | 20 |

*Note: Transitions were optimised by injecting single compound standards dissolved in solvent directly onto the mass spectrometer, reviewing the settings individually and selecting the voltages that gave the highest analyte signal.*

**Table S5:** Phase 1 chromatographic retention times (mins) and periods for the 17 pharmaceuticals. The 23 minute LC run time was divided into the following five periods: Period 1: 0-6.99 minutes (5 analytes), Period 2: 7-11.99 minutes (5 analytes), Period 3: 12-13.99 minutes (1 analyte), Period 4: 14-16.99 minutes (3 analytes) and Period 5: 17-23 minutes (4 analytes)

| **API** | **Retention time**  **(mins)** | **Period** |
| --- | --- | --- |
| 5-Fluorouracil | 1.51 | 1 |
| Allopurinol | 1.8-2.0 | 1 |
| Amantadine | 4.57 | 1 |
| Aspirin | 10.06 | 2 |
| Atenolol | 1.25 | 1 |
| Carbamazepine | 12.40 | 3 |
| Chloramphenicol | 11.85 | 2 |
| Diclofenac | 19.20 | 5 |
| Estradiol | 15.63 | 4 |
| Ethinylestradiol | 19.04 | 5 |
| Fluoxetine | 11.21 | 2 |
| Gliclazide | 15.93 | 4 |
| Ibuprofen | 18.6-18.9 | 5 |
| Indomethacin | 18.15 | 5 |
| Ketoprofen | 15.70 | 4 |
| Sulfamethoxazole | 10.44 | 2 |
| Verapamil | 10.60 | 2 |

**Table S6:** Phase 2 MRM transitions and optimised instrumental settings DP=De-clustering Potential, FP=Focussing Potential, EP= Entrance Potential, CE=Collision ‘Energy’ setting, CXP= Collision Cell Exit Potential.

| **API** | **Mode** | **Product ion (*m/z*)** | **Precursor ion (*m/z*)** | **DP**  **(V)** | **FP**  **(V)** | **EP**  **(V)** | **‘CE’ setting**  **(V)** | **CXP**  **(V)** | **Time (msec)** |
| --- | --- | --- | --- | --- | --- | --- | --- | --- | --- |
| 5-Fluorouracil | -ve | 129.0 | 86.0 | -25 | -150 | -10 | -25 | -5 | 100 |
| Atenolol | +ve | 266.4 | 225.1 | 19 | 200 | 10 | 23 | 18 | 100 |
| Estradiol | +ve | 273.0 | 255.0 | 32 | 200 | 12 | 15 | 15 | 250 |
| Ethinylestradiol | +ve | 297.4 | 279.4 | 30 | 200 | 11 | 17 | 8 | 250 |
| Ibuprofen | -ve | 204.8 | 161.0 | -20 | -120 | -5 | -10 | -15 | 100 |
| Ketoprofen | -ve | 252.8 | 208.9 | -11 | -150 | -5 | -10 | -6 | 100 |

*Note: Transitions were optimised by injecting single compound standards made up in 10% acetonitrile and 90% water directly onto the mass spectrometer, looking at the settings individually and selecting the voltage at a level that gave the highest analyte signal.Ibuprofen and ketoprofen were analysed in negative ion mode to keep the number of analytes in a period to two; however they also worked well in positive ion mode.*

**Table S7:** Phase 2 chromatographic retention times (mins) for the 6 pharmaceuticals. In positive ion mode, the two periods were 1: 0-4.001 minutes (1 analyte) and 2: 4.002-11.498 minutes (2 analytes). In negative ion mode, the two periods were 1: 0-3.5 minutes (1 analyte) and 2: 3.501-11.5 minutes (2 analytes).

| **API** | **Mode** | **Retention time (mins)** | **Period** |
| --- | --- | --- | --- |
| 5-Fluorouracil | -ve | 2.01 | 1 |
| Atenolol | +ve | 2.65 | 1 |
| Estradiol | +ve | 5.74 | 2 |
| Ethinylestradiol | +ve | 5.70 | 2 |
| Ibuprofen | -ve | 5.75 | 2 |
| Ketoprofen | -ve | 4.37 | 2 |

**9. LC-ICR-FTMS**

The same multi step gradients used in phase 1 and phase 2 for LCMS/MS analysis were also used here. MS analysis was undertaken using positive mode electrospray ionisation (ESI) - spray voltage: 4500 V, 4.5 L/min dry gas (N_2_) at 220°C and 1.6 bar nebuliser gas. The acquisition range *m/z* was 100-3000, with a transient time of 0.367 giving an estimated resolution of 66,000 at *m/z* 400. In phase 1 15 samples were run in total, five for each waste stream: one gas, one sludge and three effluent samples. In phase 2, thirty-three samples in total were analysed by LC-ICR-FTMS (six API sludge, five API ashpot, six API effluent, three API mains waster, three API Air, and one control and the blank runs used to clean the PGWTS unit between runs, which contained only Polyethylene terephthalate (PET) as a waste, for each matrix).

Active metabolites (see Table S8) were identified by examination of the accurate *m/z* values obtained in the mass spectra recorded, with an error of less than 2 ppm required to conclude that the peak was likely to be derived from the active metabolite.

**Table S8**: Transformation product parent compound and active metabolites with molecular formula

| **Parent compound** | **Active metabolite** | **Molecular formula** | **Monoisotopic mass (g/mol)** |
| --- | --- | --- | --- |
| 5-Fluorouracil | 5-fluoro-2-deoxyuridine 5’ monophosphate | C_9_H_12_FN_2_O_8_P | 326.03153 |
| Allopurinol | Oxypurinol | C_5_H_4_N_4_O_2_ | 152.033432 |
| Amantadine | - | - | - |
| Aspirin | Salicylic acid | C_7_H_6_O_3_ | 138.031693 |
| Atenolol | - | - | - |
| Carbamazepine | Carbamzepine-10, 11 epoxide | C_16_H_12_N_2_O_2_ | 252.089878 |
| Chloramphenicol | - | - | - |
| Diclofenac | 4’hydroxy-diclofenac | C_14_H_11_Cl_2_NO_3_ | 317.031728 |
| Estradiol | estrone  estriol  2-methoxyestradiol | C_18_H_22_O_2_  C_18_H_24_O_3_  C_19_H_26_O_3_ | 270.161987  288.172546  302.188195 |
| Ethinylestradiol | - | - |  |
| Fluoxetine | Norfluoxetine | C_16_H_16_F_3_NO | 295.118399 |
| Ibuprofen | 2-hydroxyibuprofen  Carboxyibuprofen | C_13_H_18_O_3_  C_13_H_16_O_4_ | 228.163255  236.104859 |
| Indometacin | - | - | - |
| Gliclazide | - | - | - |
| Ketoprofen | - | - | - |
| Sulfamethoxazole | - | - | - |
| Verapamil | Norverapamil | C_26_H_36_N_2_O_4_ | 440.267508 |

**10. Calculation of the percentage destruction of pharmaceuticals achieved by PyroPure**

**Determination of Sludge Moisture Content**

To enable comparison between the concentrations of pharmaceuticals in samples of sludge, the moisture content was determined using Equation 1. One gram of wet sludge (±0.05 g) was weighed out onto a pre-weighed foil tray (corresponds to ‘the container’ in Equation 1). Trays were placed in the drying oven at 50 °C and masses recorded every 24 hours until there was no loss of mass in three consecutive measurements. The percentage dry mass content within each sludge sample, and the total dry mass extracted from each sample for phase 1 and 2 are contained in Tables S9 and S10 respectively.

**Equation 1: Calculation of sludge moisture content**

***DM = 100*((Dry-Cont)/(Wet-Cont))***

Where:

**DM**= % of sample that is solid material

***Dry***= mass of container and dry matrix sub sample (g)

***Wet***= mass of container and wet matrix sub sample (g)

**Cont**= mass of empty container (g)

**Table S9**: Phase 1 percentage dry mass and total mass of dry sludge (g) for each run.

| **Run** | **Bulk** | | **Manufacturing** | | **Sharps** | |
| --- | --- | --- | --- | --- | --- | --- |
|  | **Dry mass (%)** | **Total dry mass (g)** | **Dry mass (%)** | **Total dry mass (g)** | **Dry Mass (%)** | **Total dry mass (g)** |
| **1** | 24.3 | 84.8 | 23.1 | 20.3 | 21.3 | 7.2 |
| **2** | 23.7 | 103.2 | 23.4 | 25.8 | 24.5 | 7.0 |
| **3** | 24.4 | 89.1 | 22.5 | 27.6 | 25.2 | 11.1 |
| **Mean** | 24.1 | 92.1 | 23.0 | 24.6 | 23.7 | 8.4 |

**Table S10:** Phase 2 percentage dry mass and total mass of dry sludge (g) for each run.

| **Run** | **Sludge** | | | **Ashpot** | | |
| --- | --- | --- | --- | --- | --- | --- |
|  | **Dry mass (%)** | **Total dry mass (g)** | **Dry mass (%)** | | **Total dry mass (g)** |  |
| **1** | 42.8 | 139.2 | 6.6 | | 4.9 |  |
| **2** | 42.8 | 81.4 | 19.8 | | 10.3 |  |
| **3** | 39.3 | 82.6 | 48.1 | | 10.1 |  |
| **Mean** | 41.6 | 101 | 24.8 | | 8.4 |  |

The concentration of each pharmaceutical determined in the liquid effluent samples was multiplied by the total volume of water used (Tables S11 and S12) to determine the total mass of API remaining in the effluent.

**Table S11:** Phase 1 - water usage in each run to the nearest litre. The overall mean was 245 L

|  | **Bulk** | **Manufacturing** | **Sharps** |
| --- | --- | --- | --- |
| 1 | 290 | 416 | 80 |
| 2 | 304 | 223 | 111 |
| 3 | 267 | 375 | 135 |
| Mean | 287 | 338 | 109 |

**Table S12:** Phase 2 - water usage in each run (litres (L)).

| Run | **water** |
| --- | --- |
| 1 | 88.40 |
| 2 | 49.32 |
| 3 | 44.70 |
| Mean | 60.81 |

**11. The assessment of chemical determinands in air emissions from Pyropure units treating pharmaceutically contaminated wastes**

Air emissions were assessed by EmCo Air Emissions limited who are a UKAS and m-CERTS accredited contractor for the assessment of particulate matter (PM_10_), nitrogen dioxide, hydrogen chloride, hydrogen fluoride, sulphur dioxide, volatile organic compounds, dioxins, furans and specific metals (mercury, cadmium, thallium, arsenic, cobalt, chromium, copper, manganese, nickel, lead, antimony, vanadium). The percentage that the Pyropure process contributed to the environmental assessment limit (EAL) following the H1 guidelines of the Environment Agency for each of the determinands was used to assess whether air emissions from treating pharmaceutical waste would pose a risk to human health or the environment see Table S13 (EA 2014).

**Table S13**: Process contribution of Pyropure to the environmental assessment limit (EAL) for particulate matter (PM_10_), nitrogen dioxide, hydrogen chloride, hydrogen fluoride, sulphur dioxide, volatile organic compounds (benzene, toluene, phenol and xylene, dioxins, furans and specific metals (mercury, cadmium, thallium, arsenic, cobalt, chromium, copper, manganese, nickel, lead, antimony, vanadium).

**
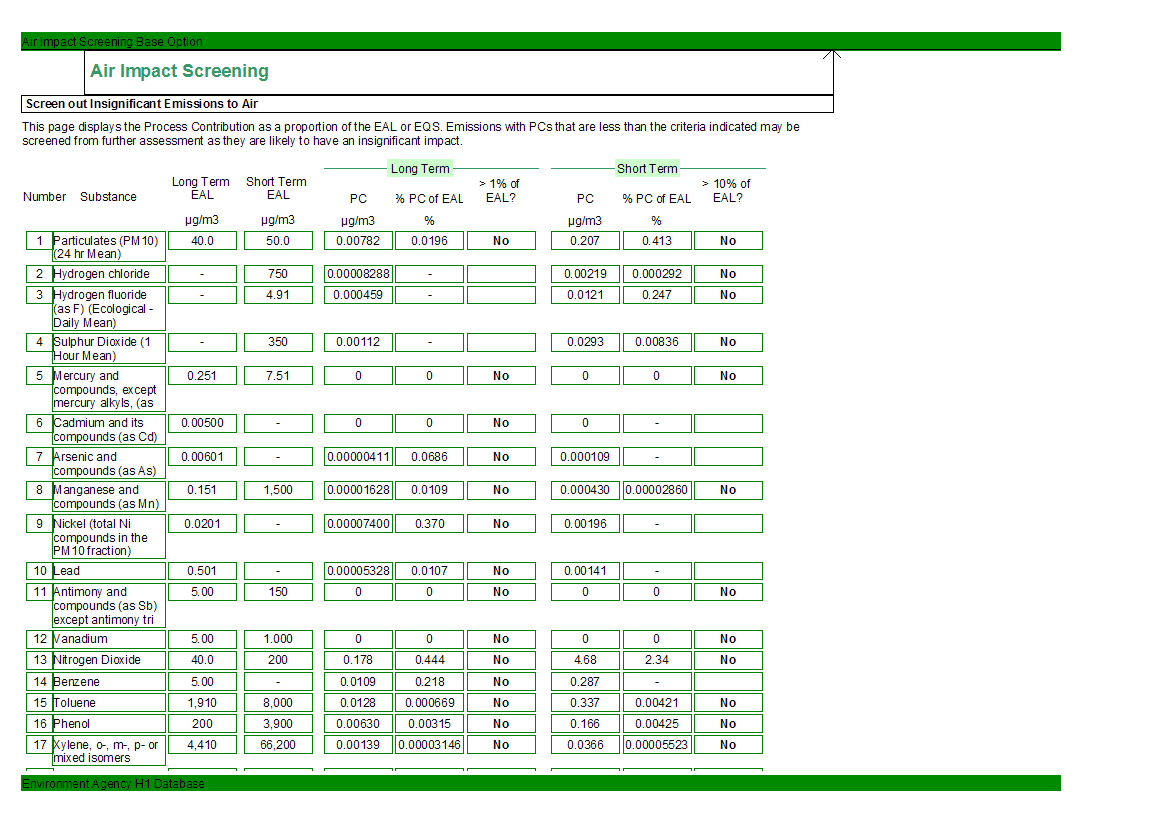
**

**12. Results: Frequency of detection**

The frequency with which pharmaceuticals were detected in the matrices analysed in phase 1 and phase 2 are presented in Tables S14 and S15. Where a pharmaceutical was detected in some, but not all of the sample, the concentration was expressed as a percentage of the added (at the start of the trials) mass of API. For example if an API was detected in two out of three samples, the average was calculated by dividing by two and not three.

**Table S14:** Percentage frequency of detection in each waste stream for each of the 17 APIs tested in phase 1

| **API** | **Bulked Waste** | | | **Manufacturing Waste** | | | **Soft Clinical/ Sharps Waste** | | |
| --- | --- | --- | --- | --- | --- | --- | --- | --- | --- |
|  | **Effluent %** | **Sludge %** | **Air %** | **Effluent %** | **Sludge %** | **Air %** | **Effluent %** | **Sludge %** | **Air %** |
| 5-Fluorouracil | - | - | - | 11  (1.64) | 100 (1.68) | 100 (1.40) | Below LOD | 100 (1.03) | 33 (0.21) |
| Allopurinol | - | - | - | Below  LOD | Below LOD | Below LOD | Below LOD | Below LOD | Below LOD |
| Amantadine | - | - | - | 66 (0.38) | 100 (0.34) | 33 (0.01) | 33 (2.83) | Below LOD | 33 (0.03) |
| Aspirin | Below LOD | † | Below LOD | Below LOD | † | Below LOD | Below LOD | † | Below LOD |
| Atenolol | - | - | - | 22 (21.95) | 100 (16.87) | Below LOD | 44 (18.25) | 100 (15.96) | Below LOD |
| Carbamazepine | - | - | - | Below LOD | Below LOD | Below LOD | Below LOD | Below LOD | Below LOD |
| Chloramphenicol | Below LOD | Below LOD | Below LOD | Below LOD | Below LOD | Below LOD | Below LOD | Below LOD | Below LOD |
| Diclofenac | - | - | - | Below LOD | Below LOD | 33 (0.03) | Below LOD | 33 (0.02) | Below LOD |
| Estradiol | - | - | - | Below LOD | Below LOD | Below LOD | 22 (35.93) | Below LOD | Below LOD |
| Ethinylestradiol | - | - | - | Below LOD | 100 (36.46) | Below LOD | 77 (7.33) | 100 (31.64) | Below LOD |
| Fluoxetine | - | - | - | Below LOD | Below LOD | Below LOD | Below LOD | Below LOD | 33 (3.46) |
| Gliclazide | - | - | - | Below LOD | Below LOD | Below LOD | Below LOD | Below LOD | Below LOD |
| Ibuprofen | 88 (0.08) | 100 (2.34) | Below LOD | 88 (1.44) | 100 (4.20) | Below LOD | 33 (5.40) | 33 (1.30) | Below LOD |
| Indomethacin | - | - | - | Below LOD | Below LOD | Below LOD | 33 (0.55 | 33 (3.77) | 33 (0.27) |
| Ketoprofen | - | - | - | 11 (1.85) | 100 (0.10) | Below LOD | 33(4.36) | 33 (0.40) | 33 (0.01) |
| Sulfamethoxazole | - | - | - | Below LOD | Below LOD | Below LOD | Below LOD | Below LOD | Below LOD |
| Verapamil | - | - | - | Below LOD | 33 (0.10) | Below LOD | 33 (0.45 | 33 (1.63) | Below LOD |

***Note:*** *Where a signal was detected, the mean concentration was converted to the percentage of the added pharmaceutical mass to which this concentration was equivalent (given in parentheses).*

† Unable to extract from sludge possibly due to degradation during extraction.

**Table S15:** Phase 2 - frequency of detection and % of added API mass that the mean concentration detected in control samples was equivalent to (i.e. the right hand column for each of liquid effluent, solids and air is the mean concentration in the samples where something was detected divided by the total mass of API added) × 100.

| API | **Effluent** | | **Sludge** | | **Air** | |
| --- | --- | --- | --- | --- | --- | --- |
|  | % freq. of detection | % of added API that the mean conc. was equivalent to | % freq. of detection | % of added API that the mean conc. was equivalent to | % freq. of detection | % of added API that the mean conc. was equivalent to |
| 5-fluorouracil | 0 | 0 | 0 | 0 | 0 | 0 |
| Ibuprofen | 33 | 0.005 | 94 | 0.057 | 0 | 0 |
| Ketoprofen | 0 | 0 | 44 | 0.00061 | 0 | 0 |
| Atenolol | 100 | 0.061 | 7 | 0.00084 | 0 | 0 |
| Estradiol | 0 | 0 | 0 | 0 | 0 | 0 |
| Ethinylestradiol | 77 | 0.16 | 56 | 0.095 | 11 | 0.041 |
| **Mean** | 33 |  | 33 |  | 1.8 |  |

**13. Control samples**

For phase 1 samples, Table S16 shows the pharmaceutical concentration detected in control samples expressed as the percentage of added API (assuming waste stream specific levels of water usage and sludge generation). Figure S2 shows the same for samples of the Pyropure site’s tapwater.

### Table S16: Equivalent percentage of API added in trials that the concentration in control samples was equivalent to

|  | **Effluent** | | | **Sludge** | | | **Air emission** | | |
| --- | --- | --- | --- | --- | --- | --- | --- | --- | --- |
|  | Bulk waste  (%) | Manufacturing waste  (%) | Soft/sharps waste  (%) | Bulk waste  (%) | Manufacturing waste  (%) | Soft/sharps waste  (%) | Bulk waste  (%) | Manufacturing waste  (%) | Soft/sharps waste  (%) |
| 5-fluorouracil | - | 0 | 0.01 | - | 0.01 | 0 | - | 0.01 | 0.01 |
| Allopurinol | - | 0 | 0 | - | 0 | 0 | - | 0 | 0 |
| Amantadine | - | 0 | 0.01 | - | 0 | 0 | - | 0 | 0 |
| Aspirin | 0.01 | 0.12 | 0.05 | 0 | 0 | 0 | 0 | 0 | 0 |
| Atenolol | - | 0 | 0 | - | 0.01 | 0.01 | - | 0.03 | 0.03 |
| Carbamazepine | - | 0 | 0 | - | 0 | 0 | - | 0 | 0 |
| Chloramphenicol | 0 | 0 | 0 | 0 | 0 | 0 | 0 | 0 | 0 |
| Diclofenac | - | 0 | 0 | - | 0 | 0 | - | 0 | 0 |
| Estradiol | - | 0 | 0 | - | 0 | 0 | - | 0 | 0 |
| Ethinylestradiol | - | 0.21 | 0.23 | - | 0.01 | 0.01 | - | 0 | 0 |
| Fluoxetine | - | 0 | 0 | - | 0 | 0 | - | 0 | 0 |
| Gliclazide | - | 0 | 0 | - | 0 | 0 | - | 0 | 0 |
| Ibuprofen | 0 | 0 | 0 | 0 | 0 | 0 | 0 | 0 | 0 |
| Indomethacin | - | 0 | 0 | - | 0 | 0 | - | 0 | 0 |
| Ketoprofen | - | 0 | 0 | - | 0 | 0 | - | 0 | 0 |
| Sulfamethoxazole | - | 0 | 0 | - | 0 | 0 | - | 0 | 0 |
| Verapamil | - | 0 | 0 | - | 0 | 0 |  | 0 | 0 |

**Figure S2:** The percentage of API that was added into the trials that was found in tap water samples in Phase 1 taken from the PyroPure factory site.

In phase 2 (Table S17), it should be noted that in the selection of control samples run on the FTMS, atenolol or ethinylestradiol did not show up in these samples but should have done at the levels detectable by this LCMS-MS system if it was there, even given the lower sensitivity of the FTMS analyses than MRM. FT-MS is a more specific measure of mass than is obtained using MRM. Tap water samples contained no detectable levels of contamination in phase 2.

Table S18 contains the active metabolites detected in control and PET samples from phase 2 run on the FT-MS. Where something was detected, the signal in the active metabolite was expressed as an approximate parent equivalent mass added to the trials.

**Table S17:** Control samples for air, mains water, liquid effluent, sludge and ashpot in phase 2 samples. The % of the amount of API added to the trials that the concentration is equivalent to and the frequency of detection is also given. Tap water samples contained no detectable levels of contamination in Phase 2.

|  |  | **5-Fluorouracil** | **Atenolol** | **Estradiol** | **Ethinylestradiol** | **Ibuprofen** | **Ketoprofen** |
| --- | --- | --- | --- | --- | --- | --- | --- |
|  |  | **Control** | **Control** | **Control** | **Control** | **Control** | **Control** |
| Air | % equivalent to | 0 | 0 | 0 | 0.0051 | 0 | 0 |
|  | frequency of detection | 0/6 | 0/6 | 0/6 | 2/6 | 0/6 | 0/6 |
| Mains water | % equivalent to | 0 | 0 | 0 | 0 | 0 | 0 |
|  | frequency of detection | 0/5 | 0/4 | 0/4 | 0/4 | 0/6 | 0/6 |
| liquid effluent | % equivalent to | 0 | 0.31 | 0 | 2.3 | 0.00015 | 0 |
|  | frequency of detection | 0/5 | 4/6 | 0/6 | 4/6 | 1/5 | 0/5 |
| Ashpot | % equivalent to | 0 | 0 | 0 | 0.0045 | 0.00071 | 0 |
|  | frequency of detection | 0/5 | 0/3 | 0/3 | 2/3 | 4/5 | 0/5 |
| Sludge | % equivalent to | 0 | 0.0039 | 0.069 | 1.96 | 0.011 | 0.00063 |
|  | frequency of detection | 0/6 | 2/6 | 2/6 | 6/6 | 3/6 | 3/6 |

|  | **Control** | | **PET** | |
| --- | --- | --- | --- | --- |
|  | **Metabolite** | **Approximate parent equivalent %** | **Metabolite** | **Approximate parent equivalent %** |
| Air | 2-hydoxyIBF  2-me | 0.016  0.033 | 2-hydoxyIBF  2-me | 0.014  0.041 |
| Mains water | 2-me | 0.036 | 2-hydoxyIBF  2-me | 0.000003  0.000001 |
| Liquid Effluent | - | - | 2-me  5-fdum | 0.000001  0.0002 |
| Solid Ashpot | - | - | 5-fdum | 0.01 |
| Sludge rep 1 | 2-hydroxyIBF  carboxyIBF  Est | 0.055  0.050  0.0019 | 2-me  carboxyIBF | 0.0000007  0.00017 |
| Sludge rep  2 | Na | Na | Na | Na |

**Table S18:** Active metabolites detected in air, mains water (i.e. water straight from the tap taken at the same time as the unit was being drained with tap water); liquid effluent; the solid ashpot residue and the sludge (solid part of the effluent); for one control run and one polyethylene terephthalate (PET) run metabolites. The active metabolites found were 5-fluoro-2-deoxyuridine 5’monophosphate (5-fdum), 2-methoxyestradiol (2-me), estrone (Est), 2-hyroxyibuprofen (2-hydroxyIBF) and carboxyibuprofen (carbIBF).

*Where an active metabolite was detected, the concentration was estimated in terms of parent equivalent and then related to the percentage of the starting mass to which this was equivalent. For control and PET and all mains water samples, no API was added and so detection must be due to background levels in the tap water.*

**References**

Environment Agency (EA) (2014) Horizontal guidelines for Environmental Permitting. Environment Agency Website <https://www.gov.uk/government/collections/horizontal-guidance-environmental-permitting>. Accessed 12 April 2015.
